# A Novel Molecular Signature Identified by Systems Genetics Approach Predicts Prognosis in Oral Squamous Cell Carcinoma

**DOI:** 10.1371/journal.pone.0023452

**Published:** 2011-08-11

**Authors:** Chien-Hua Peng, Chun-Ta Liao, Shih-Chi Peng, Yin-Ju Chen, Ann-Joy Cheng, Jyh-Lyh Juang, Chi-Ying Tsai, Tse-Ching Chen, Yung-Jen Chuang, Chuan-Yi Tang, Wen-Ping Hsieh, Tzu-Chen Yen

**Affiliations:** 1 Resource Center for Clinical Research, Chang Gung Memorial Hospital and Chang Gung University, Taoyuan, Taiwan, Republic of China; 2 Department of Otorhinolaryngology, Head and Neck Surgery, Chang Gung Memorial Hospital and Chang Gung University, Taoyuan, Taiwan, Republic of China; 3 Head and Neck Oncology Group, Chang Gung Memorial Hospital and Chang Gung University, Taoyuan, Taiwan, Republic of China; 4 Department of Nuclear Medicine and Molecular Imaging Center, Chang Gung Memorial Hospital and Chang Gung University, Taoyuan, Taiwan, Republic of China; 5 Department of Medical Biotechnology, Chang Gung Memorial Hospital and Chang Gung University, Taoyuan, Taiwan, Republic of China; 6 Department of Oral Maxillofacial Surgery, Chang Gung Memorial Hospital and Chang Gung University, Taoyuan, Taiwan, Republic of China; 7 Department of Pathology, Chang Gung Memorial Hospital and Chang Gung University, Taoyuan, Taiwan, Republic of China; 8 Department of Medical Science, National Tsing Hua University, Hsinchu, Taiwan, Republic of China; 9 Institute of Bioinformatics and Structural Biology, National Tsing Hua University, Hsinchu, Taiwan, Republic of China; 10 Institute of Statistics, National Tsing Hua University, Hsinchu, Taiwan, Republic of China; 11 Divisions of Molecular and Genomic Medicine, National Health Research Institutes, Miaoli, Taiwan, Republic of China; 12 Department of Computer Science and Information Engineering, Providence University, Taichung, Taiwan, Republic of China; AC Camargo Cancer Hospital, Brazil

## Abstract

Molecular methods for predicting prognosis in patients with oral cavity squamous cell carcinoma (OSCC) are urgently needed, considering its high recurrence rate and tendency for metastasis. The present study investigated the genetic basis of variations in gene expression associated with poor prognosis in OSCC using Affymetrix SNP 6.0 and Affymetrix GeneChip Human Gene 1.0 ST arrays. We identified recurrent DNA amplifications scattered from 8q22.2 to 8q24.3 in 112 OSCC specimens. These amplicons demonstrated significant associations with increased incidence of extracapsular spread, development of second primary malignancies, and poor survival. Fluorescence in situ hybridization, in a validation panel consisting of 295 cases, confirmed these associations. Assessment of the effects of copy number variations (CNVs) on genome-wide variations in gene expression identified a total of 85 CNV-associated transcripts enriched in the *MYC*-centered regulatory network. Twenty-four transcripts associated with increased risk of second primary malignancies, tumor relapse, and poor survival. Besides *MYC* itself, a novel dysregulated *MYC* module plays a key role in OSCC carcinogenesis. This study identified a candidate molecular signature associated with poor prognosis in OSCC patients, which may ultimately facilitate patient-tailored selection of therapeutic strategies.

## Introduction

Oral cavity squamous cell carcinoma (OSCC) is the sixth most common cancer worldwide and affects approximately 405,000 people each year (www-dep.iarc.fr). OSCC mainly affects males who are exposed to various oral risk factors, including tobacco smoking, betel quid chewing, or alcohol consumption. In most South Asian countries, approximately 85% of all patients with OSCC habitually use betel quid.

In endemic betel quid chewing areas, researchers have associated numerous clinical and pathological variables with adverse prognosis in OSCC [1], [2], [3], [4], [5], [6], [7]. Considering this, an evidence-based assessment of risk factors in OSCC requires a detailed pathological examination to gauge prognostic features such as extracapsular spread (ECS), pathologically-positive nodes, and tumor depth, as well as accurate estimates of other aspects which constitute important extrinsic disease modifiers. However, traditional risk factors for individual prognostication have limited value because patients with tumors of the same clinicopathological features have heterogeneous responses to treatment. Importantly, there are currently limited data available on the genetic modifiers of clinical outcomes in OSCC. In endemic betel quid chewing areas, previous research has identified several genes as potentially associating with OSCC progression, including *TP53*, *CCND1*, *EGFR*, *ETS1*, *RB1*, *VEGF*, and *STAT3* [8], [9], [10]. Unfortunately, most common gene variants have insufficient prognostic value to be used in clinical practice and single-gene strategies are prone to bias and incompletion. The explosion of genomic data enabled the discovery and validation of candidate DNA markers and dysfunctional gene modules for predicting prognosis. For example, there are studies specifically addressing the role of copy number variations (CNVs) in OSCC [11]. The identified chromosomal alterations included gains of chromosomes 3q, 5p, 8q, 12p, 20 and X as well as losses of chromosomes 3p, 4 ,8p, 13q, 17p and 18q [12], [13], [14]. However, the phenotypic effects of CNVs are likely to be neutral. Some CNVs do not necessarily harbor genes that drive the disease pathogenesis [15]. Thus, to identify disease-related variants, other studies have demonstrated the potential of integrating DNA variations and gene expression levels [15], [16], [17], [18]. In a total of 20 OSCC samples, Xu et al. have identified that the 11q13.2-q13.3 region contains genes which show a high degree of correlation between copy number and gene expression [17]. However, this and other previous studies typically had small sample sizes. The relative contribution of CNVs to whole-genome expression pattern and clinical outcomes has yet to be systematically evaluated. In order to identify reliable and clinically-useful biomarkers, the present study dissected the genetic components of gene expression in OSCC, on a large scale, with the goal of identifying the disease susceptibility variants as well as disease-responsive gene modules.

In this study, the increased expression of genes located in amplified regions supported the predicted copy number amplifications. Fluorescence *in situ* hybridization, in a validation panel consisting of 295 cases, confirmed their clinical significance. Assessments also included analyses of the set of CNVs with genome-wide expression profiles in order to investigate whole-genome transcriptional changes in response to the unstable genomic regions. Next, the results of systems genetic studies were examined in relation to clinicopathological and prognostic features. The final part of the research consisted of a functional study using the manually curated molecular interaction network. The overall findings of the present investigation have implications for prognostication and may ultimately facilitate patient-tailored selection of therapeutic strategies in OSCC.

## Results

### Genome-wide detection of CNVs in OSCC specimens

**Figure 1:**
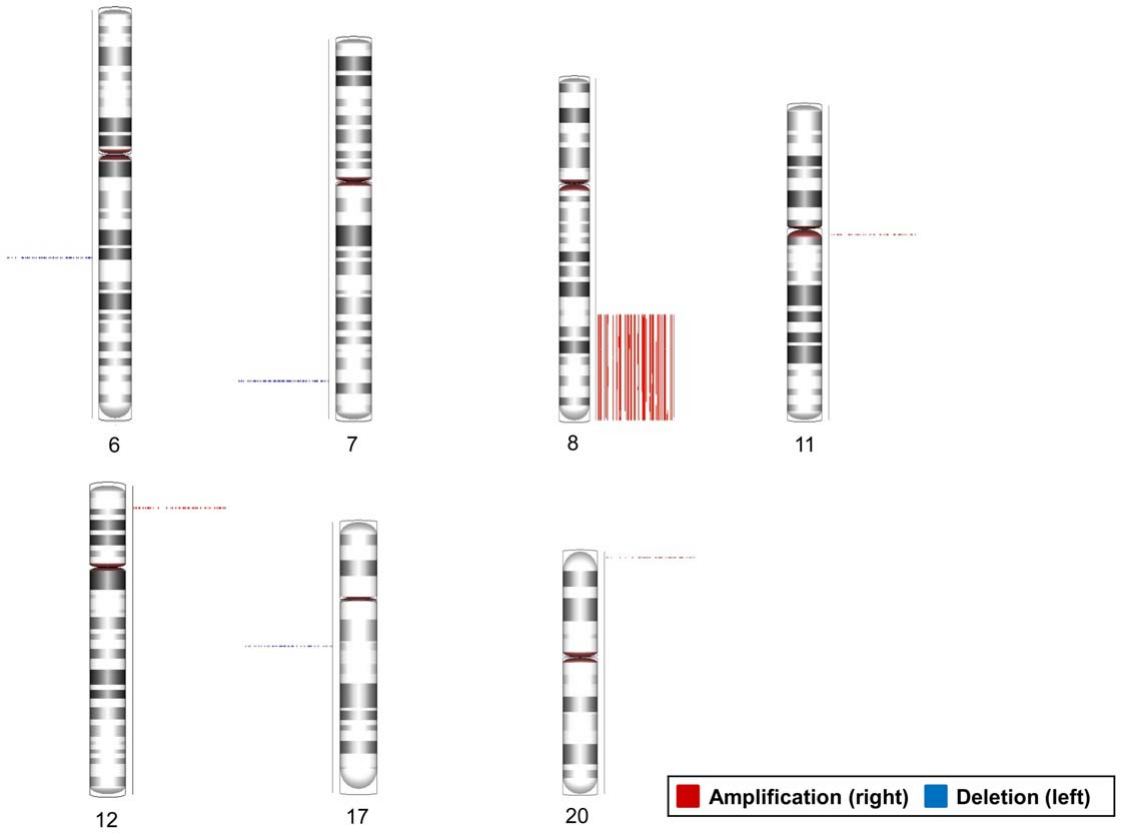
Genomic loci of the common CNVs occurred in at least 40 OSCC samples. The copy number state of each patient is reported as a column placed to both sides of each chromosome. In the right side of each chromosome, red lines denote amplifications while blue lines on the left indicate deletions. The empty columns indicate the patients with unchanged copy number of these loci.

The computational methods described in the Materials and Methods section detected individual CNVs from each OSCC patient. The histogram in Figure S1 summarizes the distribution of the CNV lengths. Most CNVs were rare and present in a few patients only, possibly indicating relatively minor effects on OSCC carcinogenesis. Thus, this study initially focused on the common CNVs detected in more than 30% of the OSCC patients, then evaluated if any of the common CNVs has important clinical effects on the management of OSCC patients. This narrowed the list of CNVs to 83 common CNVs occurring in at least 40 patients. The common gains occurred in chromosomes 8q22.2∼24.3, 11q11, 12p13.31, and 20p13; the common losses occurred in 6q16.3, 7q34, and 17q21.2. Of the 83 common CNVs, 66 located on chromosome 8q (Figure 1).

Common CNVs are known to also occur in the general population. Searching the Database of Genomic Variants (DGV) [19] for the 83 common CNVs identified that 22 of the 83 CNVs are general polymorphisms in healthy people. The remaining 61 CNVs are all on chromosome 8q22.2∼24.3 and show little or no overlap with DGV entries (Table S1), indicating that the 61 common CNVs are not pervasive in healthy subjects. The patient sets affected by each of the 61 CNV regions were highly overlapped and comprised only a slightly different set of OSCC patients. The union set included 46 patients. The OSCC patients were hence grouped into amplified (n = 46) and non-amplified (n = 66) sets.

Fluorescence in situ hybridization (FISH) of the MYC gene, in a replication panel consisting of 295 cases, supported CNVs results in the 8q24 region. The proto-oncogene *MYC* is located in the study's predicted amplified regions. *MYC* regulates the expression of a number of genes involved in angiogenesis, cell growth, proliferation, differentiation, apoptosis, and cell cycle progression [20] so changes in its expression can be amplified among downstream genes. It can also interact with the pre-replicative complex and cause the activation of unscheduled and unstable replication origins [21], leading to long-range DNA amplification [20], [21], [22]. FISH experiments identified 42 amplified cases which shared similar clinicopathological traits with computational predictions as detailed in the following section.

### Association of CNVs with Clinical Traits

**Figure 2:**
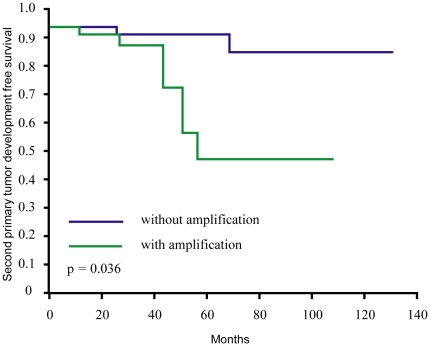
Kaplan-Meier estimates for the development of second primary tumors according to the presence or absence of 8q22.2∼24.3 amplifications (log-rank test, p = 0.036).

To investigate the clinical significance of the common CNVs on 8q22.2∼24.3, the patients were divided into two subgroups with (n = 46) and without amplification (n = 66). These CNVs significantly associated with death within five years (odds ratio = 2.412, p = 0.0249), disease-specific death within five years (odds ratio = 2.500, p = 0.0246), and ECS (odds ratio = 2.400, p = 0.028, Table 1). In addition, Kaplan-Meier estimates revealed that the patients with amplification have a shorter event-free time in terms of development of second primary malignancies with a p value of 0.036 for the log-rank test (Figure 2). In the validation panel, FISH supported the results concerning the predictive value of 8q22.2∼24.3 amplification for ECS (p = 0.002) and overall survival (p = 0.004). The association with the development of second primary tumors was of borderline statistical significance (p = 0.057), suggesting a moderate effect exists.

**Table 1:** Significant association of 8q22.2∼24.3 amplification with survival rates and pathological characteristics.

	Death within 5 years		Disease-specifc death within 5 years		Extracapsular spread	
	OR*	p	OR	p	OR	p
	(95% CI§)		(95% CI)		(95% CI)	
**Amplification on 8q22.2∼24.3**	2.412	0.0246	2.500	0.0246	2.400	0.028
	(1.109, 5.245)		(1.114, 5.610)		(1.091, 5.279)	

*OR: odds ratio.

§ CI: confidence interval.

### Effects of 8q22.2∼24.3 amplifications on gene expression

**Figure 3:**
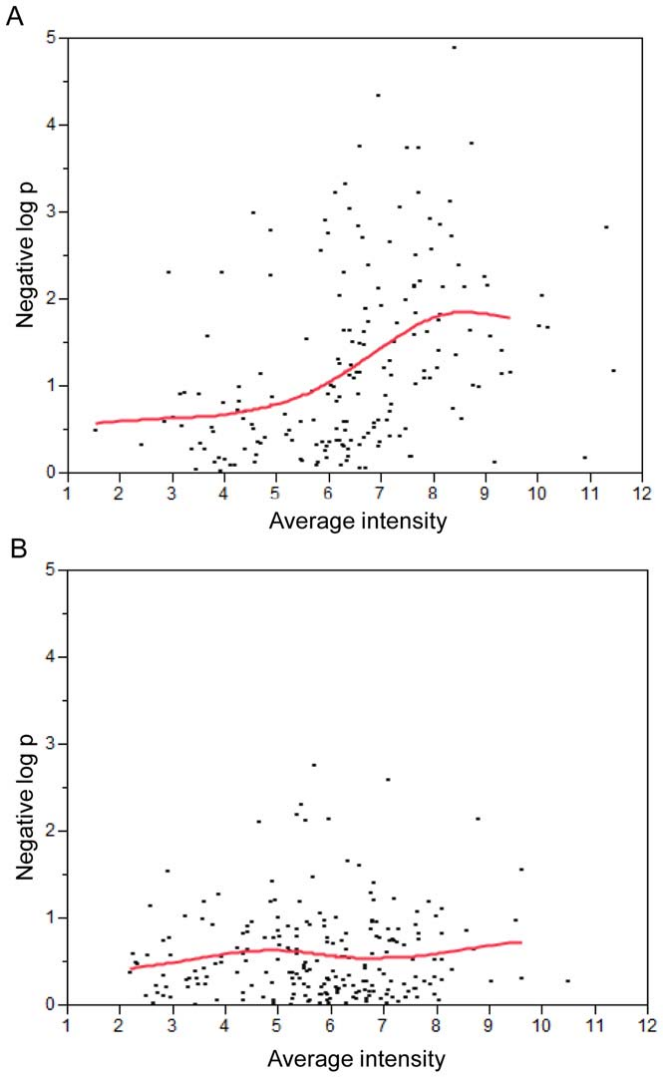
Expression patterns of genes located within and outside the amplified regions. The negative log10 p values derived from *t*-tests were plotted against the average intensity levels for (A) genes located within and (B) outside the amplified regions. The spline curves were fit to extract the main trend of the data.

In the present study, Affymetrix Human Exon 1.0 ST Array was used to obtain the gene expression patterns for the 184 well-annotated genes within the 61 common CNVs, which scattered from 8q22.2 to 8q24.3. A simple *t*-test compared the amplified (n = 28) and non-amplified (n = 29) samples, which were typed (profiled) with both SNP and Exon arrays. Comparison of the p values obtained from the *t*-tests with another 219 well-annotated genes located on chromosome 8 and outside the amplified regions showed that genes located within the common amplicons have enhanced significance levels of the expression change compared with genes in the non-amplified regions (Figure S2). Plotting the expression levels against the significance of the changes facilitated the identification of a gene dosage effect (Figure 3). For the genes within the common amplicons, the significance level roughly increased with the expression levels (Figure 3A). Assuming that genes with functional roles in oral tissue have higher expression levels, these genes should demonstrate a dosage effect and increased likelihood of changes to expression in response to sequence variations. In contrast, genes located outside of the predicted CNVs produce only random patterns (Figure 3B). The distribution of p values was highly homogeneous across the entire spectrum of gene expression levels. Changes in expression levels were mostly consistent with predictions based on gains of specific DNA segments.

Among the various phenotypes, gene expression acts as an intermediate phenotype situated between CNVs and other clinical phenotypes. This study, therefore, integrated gene expression profiles to assess the influences of the CNVs on genome-wide variations in gene expression and elucidate the molecular mechanisms behind the observed associations between CNVs and clinicopathological features.

To this end, the gene expression profile analyses focused on identification of the expression traits which responded to each of the 61 common CNVs. eCNV denoted the set of CNVs associated with expression traits. The eCNV can be in proximity of a gene whose expression is regulated by the eCNV itself; or it can regulate the expression of a nearby gene which, in turn, has regulatory effects on several distant genes. The eCNVs are also considered as the genetic components of gene expression [23] underlying oral cancer carcinogenesis.

The significant associations between CNVs and OSCC outcomes suggest that expression changes in specific genes within these amplicons might be critical to OSCC progression. If an eCNV has an effect on expression of the genes located herein, the eCNV is defined as cis-eCNV. Of the 184 well-annotated genes located within the 61 CNVs, 24 had strong copy number dosage effect with respect to the 16 cis-eCNVs that enclose those genes (Table 2). These 16 cis-eCNVs are deemed high confidence predictions. Such 16 cis-eCNVs and the 24 genes represent good candidates for the associated phenotypic variation.

**Table 2:** List of 16 CNVs and 24 genes with strong cis- control signals.

Index	CNV start position	CNV end position	Genes with strong cis- control		
**1**	101000476	101348744	***POLR2K***		
**2**	102634362	104468714	***FZD6***		
**3**	104473220	105045989	***WDSOF1***	***RIMS2***	
**4**	105045989	106820189	***LRP12***	***RIMS2***	
**5**	109574950	110915943	***NUDCD1***		
**6**	117702319	119455900	***EXT1***	***MED30***	***UTP23***
**7**	119717186	121345471	***TAF2***	***DEPDC6***	
**8**	121345471	122332581	***MRPL13***		
**9**	123273590	126370411	***NDUFB9***		
**10**	139583045	141890601	***EIF2C2***	***CHRAC1***	
**11**	139583045	142231340	***PTK2***		
**12**	143461547	144770188	***LY6K***	***PYCRL***	
**13**	144784148	144995051	***ZNF707***	***PUF60***	
**14**	144995051	145163601	***GRINA***		
**15**	145163601	145986967	***HSF1***		
**16**	145986967	146268960	***COMMD5***	***C8orf33***	

Notably, the 24 candidate genes were differentially expressed between tumors and normal tissues, as well as between patients with and without amplifications of the 16 *cis*-eCNVs elements. Previous research has shown that 11 out of the 24 genes relate to OSCC carcinogenesis and 11 out of the remaining 13 genes link to other cancers such as prostate, breast, and colorectal cancer (Table S2). However, these previous research did not clarify the extent to which these genes influence cancer prognosis.

### Association of the 24 amplified genes with clinical traits

To understand the prognostic effects of the 24 genes on clinical phenotypes, this study investigated the association of the 24 genes with clinical outcomes using Cox regression analysis (Table 3) and also evaluated the pairwise interactions among the 24 genes (Table S3). Results indicated that overexpression of *EIF2C2* significantly associated with an increased risk of developing second primary tumors. Survival outcomes associated with the expression of *PTK2* through its interactions with other genes, such as LRP12 or GRINA (Table S3). Moreover, this study, for the first time, demonstrated *MED30* and *DEPDC6* as relapse-related genes which significantly associated with an increased risk of tumor relapse, including local recurrence, neck lymph node metastasis, and distant metastasis. The mechanism by which DEPDC6 and MED30 promote tumor relapse warrants further investigation.

**Table 3:** Cox regression analysis of different time-to-event clinical traits with the 24 selected genes.

Risk factor	Predictor	OR (95% CI)	p
**Disease-specific survival**	*LRP12*	2.244 (1.004, 5.016)*	0.047
**Second primary tumors**	*NUDCD1*	0.193 (0.039, 0.948)	0.043
	*EIF2C2*	38.816 (1.979, 761.385)	0.016
**Local relapse**	*MED30*	11.555 (1.446, 92.345)	0.021
**Neck relapse**	*TAF2*	0.313 (0.111, 0.879)	0.027
**Local+neck relapse**	*DEPDC6*	1.755 (2.399, 70.354)	0.000
	*MED30*	12. 992 (1.279, 2.407)	0.003
**Distant metastasis**	*ZNF707*	17.000 (1.676, 172.379)	0.017
	*MED30*	14.147 (1.305, 153.384)	0.029
**Relapse (local+neck+distant metastases)**	*DEPDC6*	1.454 (1.054, 2.007)	0.023
	*MED30*	9.350 (2.038, 42.888)	0.004

### Enrichment Analysis for the Transcriptional Module

Since the common amplicons in 8q22.2∼24.3 have significant prognostic effects, it is important to clarify whether the CNV-associated genes are functionally relevant and how they related. As known, a CNV can control expression traits in *cis* or *trans* form (see Materials and Methods). Thus, in addition to the 24 *cis*-control genes described previously, this study included the other 61 *trans*-control genes with expression levels highly associated with the 16 high-confidence CNVs and located on any chromosome. The 85 CNV-associated genes should provide a more complete picture of the regulatory map (Table S4). We explored their common upstream regulators by enrichment analyses of the transcriptional modules and gene ontology based on a manually curated database. A transcriptional module consists of co-regulated genes and a common transcription factor regulating their expression. Ultimately, the aim was to determine if the 85 genes can be clustered in specific functional groups.

**Figure 4:**
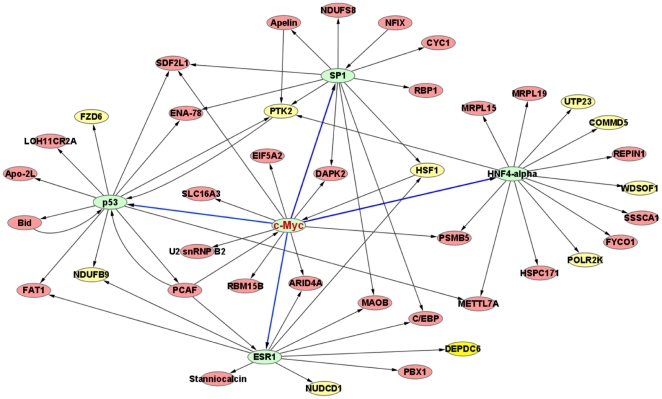
*MYC*-centered regulatory network for the CNV-associated genes in OSCC. The green nodes indicate the top five influential transcriptional factors. The *MYC* regulates the other four transcription factors (blue arrows). Yellow nodes denote *cis*-control genes while red nodes represent *trans*-control genes (only the top five transcriptional modules were plotted).

The enrichment analyses were performed using the hypergeometric test. The top two transcriptional modules are controlled by *HNF4A* and *SP1*, which bind to promoter sites of genes involved in cell proliferation [24], [25]. The later three transcriptional modules are controlled by *MYC*, *P53*, and *ESR1*, which have been implicated in G1/S transition [26], [27], [28]. The p values for the top five transcriptional modules ranged from 5E-37 to 9.21E-23. Analyses also included searching the enriched GO terms for genes in each module (Table S5). Of the top five transcription factors, the *MYC* gene is located in 8q24 amplified region. Concordantly, findings included significant enrichment of the *MYC* transcriptional module, and *MYC* was also found to regulate other transcription factors, *HNF4A*, *SP1*, *P53*, and *ESR1*, of other top five transcriptional modules (Figure 4). *MYC* also appeared to influence 14 out of the 24 *cis*-control genes through other transcription factors (Figure S3). Notably, not only *MYC* itself but also the copy numbers of the 24 genes were co-amplified. Most of these amplified genes were enriched in the *MYC* module. The more aggressive phenotypes of OSCC tumors are probably the consequences of multiple deregulations of the multiple copy number amplified genes in the *MYC* module. This study is the first to implicate the *MYC*-centered regulatory network in OSCC susceptibility (Figure 4 and Figure S3).

## Discussion

Part of our reported copy number amplifications confirmed the previous results with clinical evidence [12], [29], [30], [31]. The current study expanded the understanding of 8q region in relation to cancer etiology, with more accurate identification of CNV-associated genes. Its main findings are summarized and discussed in detail below.

Firstly, this study identified that DNA amplifications scattered from chromosome 8q22.2 to 8q24.3 significantly associate with an increased risk of ECS, poor survival, and possible early development of second primary tumors. OSCC patients have a higher risk of developing a second primary tumor than patients with other cancers. Second primary tumors and ECS are two important risk factors which limit patients' survival. The present study's results, therefore, potentially compensate for the lack of molecular markers for predicting the risk of ECS and second primary tumors.

Secondly, to estimate the overall effects of CNVs on gene expression *in vivo*, this study conducted association analyses of CNVs with genome-wide gene expression levels, identifying a total of 85 CNV-associated genes. Expression changes in 24 of these genes significantly associated with distant metastases, tumor relapse, the development of second primary tumors, and poor survival outcomes. Among the 24 genes, EIF2C2 encodes for a catalytic component of RNA-induced silencing complex which modulates the efficacy of microRNAs. Findings of an association between *EIF2C2* expression and the risk of development of second primary tumors may prompt further studies aimed at identifying specific microRNAs and their targets in OSCC cells. To our knowledge, this study also described for the first time other genes (e.g., MED 30 and DEPDC6) as biomarker candidates for tumor relapse. The *MED30* is a component of *TRAP*/Mediator complex, which is required for both basal and activator-dependent transcription [32]. Recent reports have demonstrated that the number of *MED30* copies is also amplified in breast cancer [33]. As for DEPDC6, it encodes a *MTOR* inhibitor and is also negatively regulated by *MTOR* [34], [35]. Of the 24 genes with strong dosage effects, *DEPDC6* was the only one to show a negative gene dosage effect. This finding agrees with previous research which demonstrated that feedback regulation of the *MTOR* signaling pathways ultimately represses *DEPDC6* expression [34], [35], [36]. The mechanisms whereby the 24 genes cooperatively determine the clinical phenotypes have yet to be fully elucidated. However, the enrichment studies of the 85 CNV-associated genes on the transcriptional modules provide clues to clarify this issue. The 24 identified genes may be involved, at least in part, in cell growth and cell cycle regulation.

Thirdly, another important point raised by this study is the establishment of a master regulator located upstream of the 85 CNV-associated genes. Findings first suggest that the *MYC*-centered regulatory network may explain the observed differences in clinical outcomes of OSCC patients instead of direct consequences of MYC itself. The *MYC* gene can regulate a number of different transcription factors, including *HNF4A*, *SP1*, *P53*, and *ESR1* [37], [38], [39], [40], which comprise this study's top five transcriptional modules. This may imply the combinatorial function of transcription factors in *MYC* module in OSCC tumors. Analyses also revealed concordant associations between the deregulated expression traits and the amplified *MYC* locus. Taken together, these results highly suggest that *MYC* may act as an upstream master regulator of the OSCC susceptibility genes. In the validation panel of 295 cases, the amplification of *MYC* also significantly associated with ECS status, tumor relapse, and survival. However, MYC alone has less prognostic impact on clinical outcome. As the mechanisms underlying OSCC carcinogenesis are multifactorial, *MYC* alone may not sufficiently precipitate a dysregulated state in gene expression. Since most of the 24 copy number amplified genes were enriched in the MYC module, deregulations of multiple co-amplified members collectively induce more CNV-associated genes in the MYC module to be dysregulated. This may prompt speculation that beyond MYC itself, multiple copy number amplified genes and abundant dysregulated genes in the *MYC* module may ultimately lead to dysfunction of the cell cycle, cell proliferation, and invasion in OSCC tumors. The ability of the MYC module to reprogram somatic cells into stem cell-like cells may explain its association with carcinogenesis [41].

Finally, of much interest and of most clinical relevance, the study findings clearly suggest that CNVs within the 8q22.2∼24.3 region may ultimately facilitate patient-tailored selection of follow-up and treatment strategies. This may especially apply in low- to moderate- risk OSCC patients with no evidence of ECS, who should change their treatment modalities to more aggressive approaches in the presence of prognostic molecular biomarkers of adverse outcomes. The association of CNVs within the 8q22.2∼24.3 region with the risk of ECS or second primary tumors may also lead to a more intensive follow-up schedule in patients bearing adverse prognostic biomarkers, but who are initially negative for ECS or second primary tumors. In this group of high-risk patients, the ultimate goal is to improve the detection of potentially salvageable recurrent lesions. According to our integrated analyses, the 24 genes located within the amplified regions provide promising candidates for explaining the different clinical phenotypes of OSCC, as well as predicting prognosis in this clinical entity.

In summary, the prognostic stratification of patients based on our newly identified molecular signature may lead to significant changes in treatment modalities, shifting from conservative to more aggressive approaches for patients reclassified as being high-risk. These findings are important for devising individualized follow-up strategies based on the presence or absence of the molecular signature. Our study clearly demonstrates the potential for clinical application and provides a framework for future studies. However, while this study's findings may have elucidated some issues regarding OSCC carcinogenesis, caution should be exercised before generalizing from these results. Large scale assessments of clinical outcomes in OSCC patients with the use of the molecular signature are of utmost importance. Overall, this study's results may directly facilitate progress in the clinical management of OSCC patients.

## Materials and Methods

### Ethics Statement

Written informed consent was obtained from all participants. This study was approved by the Chang Gung Memorial Hospital Human Research Ethics Committee (Protocol number 98-3645B).

### Patients and Specimens

OSCC tissues from 112 patients (Table S6) and 24 non-cancerous controls were collected from January 2000 to December 2008. Each sample was diagnosed histologically. All 112 OSCC samples and 10 non-neoplastic tissues were examined using the Affymetrix SNP Array 6.0 platform. A total of 57 OSCC samples and 22 non-cancerous controls were analyzed using the Affymetrix Exon 1.0 ST array. In total, 57 neoplastic and eight non-neoplastic specimens were examined using both arrays. The results on CNVs were confirmed using fluorescence *in situ* hybridization (FISH) in a validation panel consisting of 295 unrelated OSCC cases.

### SNP typing and transcriptome profiling

Tissue was homogenized in a homogenizing buffer (50 mM Tris-HCl [pH 8.2], 20 mM EDTA, 10 mM NaCl and 1 mg/ml proteinase K) incubated at 56°C overnight. DNA was purified by phenol/chloroform extraction and sodium acetate/ethanol precipitation. DNA was hybridized to an Affymetrix Genome-wide Human SNP Array 6.0 (Affymetrix, Santa Clara, CA, USA) according to the manufacturer's protocol. Total RNA was isolated from tissue using the Qiagen RNeasy Mini Kit (Qiagen, Germantown, MD, USA) according to the manufacturer's protocol. Quality of RNA was ensured by Bioanalyzer 2100 (Agilent, Santa Clara, CA, USA). RNA from these preparations was used to probe the Affymetrix GeneChip Human Gene 1.0 ST Array according to the Affymetrix standard protocol. The data are publicly available in the Gene Expression Omnibus database under the accession number GSE25104.

### Copy Number Variations

Quality control for SNP arrays was performed at the sample level. The average Birdseed call rate [42] was 99.5%, with a minimum call rate of 96.3%. CNVs were scored using the Hidden Markov Model (HMM) and the segmentation approaches available within the Partek software package 6.5 (Partek Incorporated, St Louis, MO, USA). The signal intensity of each tumor was compared with the pooled intensity of the reference calculated as the mean of all non-cancerous samples for each SNP probe set. HMM-detected regions were considered as CNVs if they consisted of at least 10 consecutive imbalanced SNPs. Copy number variations occurring in at least 40 of the 112 tumor samples were considered as common (>30%) and were selected for further analysis.

### Association between CNVs and Gene Expression

A total of 57 neoplastic and eight non-neoplastic specimens were examined using both arrays. The tumor samples were divided into three groups based on the copy number status (amplification, deletion, no change) at each CNV locus. However, all the 83 regions identified in this study showed only two distinct states. Therefore, two-sample *t*-tests were used to assess the association between the expression level and the 83 common CNVs. The nominal p values were adjusted for multiple testing using the false discovery rate (FDR) procedure, and a standard threshold of 5% was applied for declaring significance. Using these criteria, 148 expression traits (0.836%) on the array were found to associate with at least one common CNV. Significant predictors were denoted as eCNVs, in keeping with the concept of eQTL in the literature [18]. In this study, the *cis*-regulatory effect results from the CNV segment that directly influences transcript levels of genes in that segment, and the *trans*-regulatory effect alters gene expression outside the CNV segment. Of the 148 expression traits, 24 associated with 16 CNV regions harboring the gene itself. The 16 CNV regions were termed *cis*-eCNV in the context and also controlled additional 61 of the 148 genes based on the t-test. The 61 genes do not locate within the 16 CNVs and were, thus, considered as being under *trans*-control.

### Fluorescence in Situ Hybridization

Fluorescence *in situ* hybridization (FISH) technologies were used to confirm *MYC* amplification within the 8q24 region. FISH was performed on tissue microarray sections. Tissue sections (4 µm thick) were mounted onto coated slides, air-dried, and baked overnight at 56°C. They were then deparaffinized in xylene and 100% ethanol, treated with paraffin pretreatment solution (Paraffin Pretreatment Kit II, Abbott Molecular/Vysis, Des Plaines, IL, USA) for 10 min at 80°C, washed with distilled water, and treated with protease for 15 min at 37°C. After washing in distilled water, the slides were dehydrated in gradient alcohol/water mixtures. Probe mixture (10 µL) was applied to the slides in an approximately 4 cm^2^ area selected in a pure tumor cell population. The slides were denatured for 5 min at 80°C and hybridized for 16 hr at 37°C in a ThermoBrite™ hybridizer (Abbott Molecular, Des Plaines, IL, USA). The excess probe was washed away using 2× saline sodium citrate buffer (SSC)/0.3% NP-40 at 72°C for 2 min, and the nuclei were counterstained with 4′,6′-diamidino-2-phenylindole dihydrochloride (DAPI)/Vectashield (Vector Labs, Burlingame, CA, USA). For scoring, the tissue sections were examined under a fluorescent microscope (Olympus BX60, Southall, Middlesex, UK) operated using DP software (Olympus). Hybridization signals from at least 60 cells were scored to assess the average copy number status. Amplification was considered to be present in all cases showing >1.5-fold amplification compared with chromosome 8 centromere.

### Association of CNVs and Gene Expression Profiles with Clinical Pathological Traits

The association of 226 clinicopathological traits with CNVs and expression profiles were examined. The main traits included the presence of ECS, pathological neck lymph node metastases, perineural invasion, vascular and lymphatic invasion, tumor depth, five year local recurrence, neck recurrence, distant metastasis, second primary tumors, disease-specific survival, and overall survival. The chi-square test was used to determine the associations between binary clinical outcomes and CNVs. Survival analysis in relation to CNVs was performed using the log-rank test and demonstrated using Kaplan-Meier curves. Cox regression was applied to analyze the independent associations of the 24 differently expressed genes with clinical outcomes. Both main effects and pairwise interactions were considered. All statistical analyses were performed using R (www.r-project.org) and the SPSS software package 16.0 for Windows (SPSS Inc., Chicago, IL, USA).

### Enrichment Analysis for the Transcriptional Module

Enrichment analysis was performed using Meta-Core analytical suite version 6.3 build 25485 (GeneGo, St Joseph, MI, USA; http://www.genego.com). The software is based on a manually curated database of molecular interactions, canonical pathways, and knowledge-based ontologies of cellular processes [43]. Enrichment analysis on the 85 genes associated with the 16 high-confidence CNV regions was performed using the Analyze Networks function in the MetaCore. This algorithm generates transcriptional modules centered on transcription factors. Each transcriptional module was scored based on the probability of creating modules with a higher than random saturation for all the 85 genes. The genes were also evaluated for the enrichment of the Gene Ontology (GO). The significance of both enrichment analyses was calculated based on the hypergeometric distribution.

## Supporting Information

Figure S1 **Distribution of CNV (n = 3,838) sizes identified in 112 OSCC specimens (mean size = 3,915 kb; median size = 66 kb).**
(TIF)

Figure S2 **The negative log10 p values derived from** ***t*** **-tests were plotted against the physical position on chromosome 8.** The genes located within the predicted amplification regions on 8q22.2∼24.3 demonstrated increased significance levels compared with the nonamplified regions.
(TIF)

Figure S3 **Nodes with red circles indicate the 14 genes influenced by cMyc through other transcription factors.** Nodes with blue circles denote the top five master transcription factors other than cMyc. In our genetic component of gene expression analysis, the expression levels of 10 (black circles) of the 24 cis-control genes were predicted to be affected by the amplified cMyc locus. The results of genetic component of gene expression analysis were in agreement with enrichment analysis of transcriptional modules.
(TIF)

Table S1 **The 83 common CNVs. Each of these occurred in at least 40 OSCC patients.**
(DOC)

Table S2 **Associations of the 24 amplified genes with different cancers according to published literature.**
(DOC)

Table S3 **Cox regression analysis of different time-to-event clinical traits and the 24 selected genes, as well as interactions between the 24 genes.**
(DOC)

Table S4 **85 CNV-associated genes.**
(DOC)

Table S5 **Enrichment analyses of transcriptional modules and Gene Ontology processes for the 85 genes associated with the 16 high-confidence CNV regions.**
(DOC)

Table S6 **General characteristics of the study participants.**
(DOC)
