# Supplementary figures and images for: A Novel Molecular Signature Identified by Systems Genetics Approach Predicts Prognosis in Oral Squamous Cell Carcinoma
Source: PLoS One. 2011 Aug 11;6(8):e23452. doi: 10.1371/journal.pone.0023452 (PMC3154947; doi:10.1371/journal.pone.0023452)

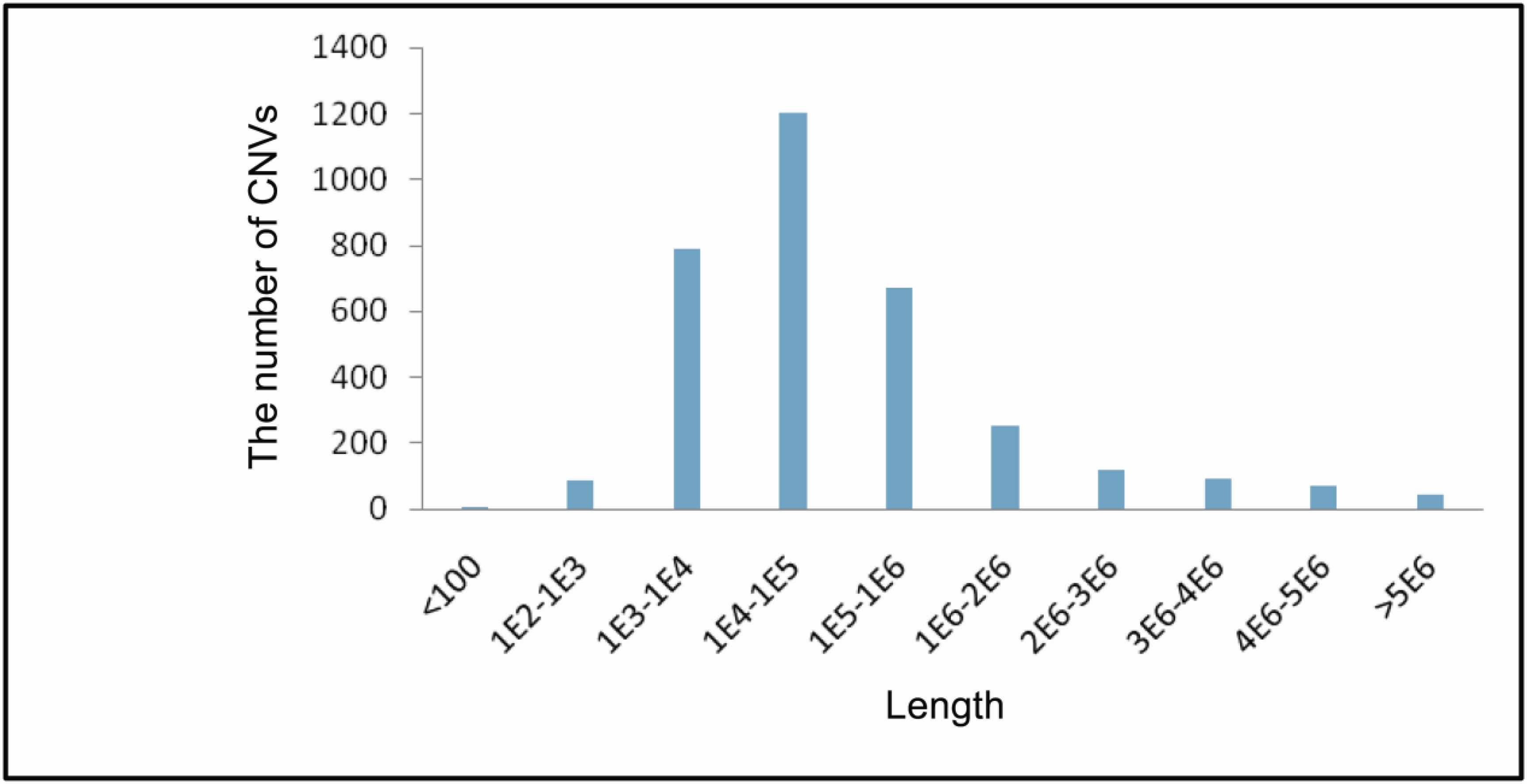

Supplement: Figure S1 — Distribution of CNV (n = 3,838) sizes identified in 112 OSCC specimens (mean size = 3,915 kb; median size = 66 kb). (TIF) [file pone.0023452.s001.tif]

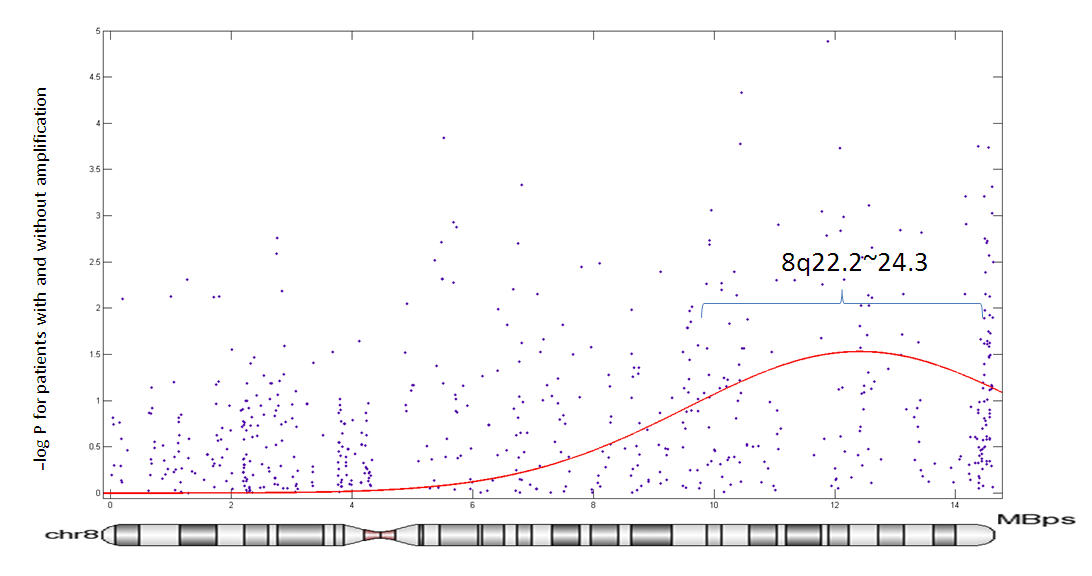

Supplement: Figure S2 — The negative log10 p values derived from t -tests were plotted against the physical position on chromosome 8. The genes located within the predicted amplification regions on 8q22.2∼24.3 demonstrated increased significance levels compared with the nonamplified regions. (TIF) [file pone.0023452.s002.tif]

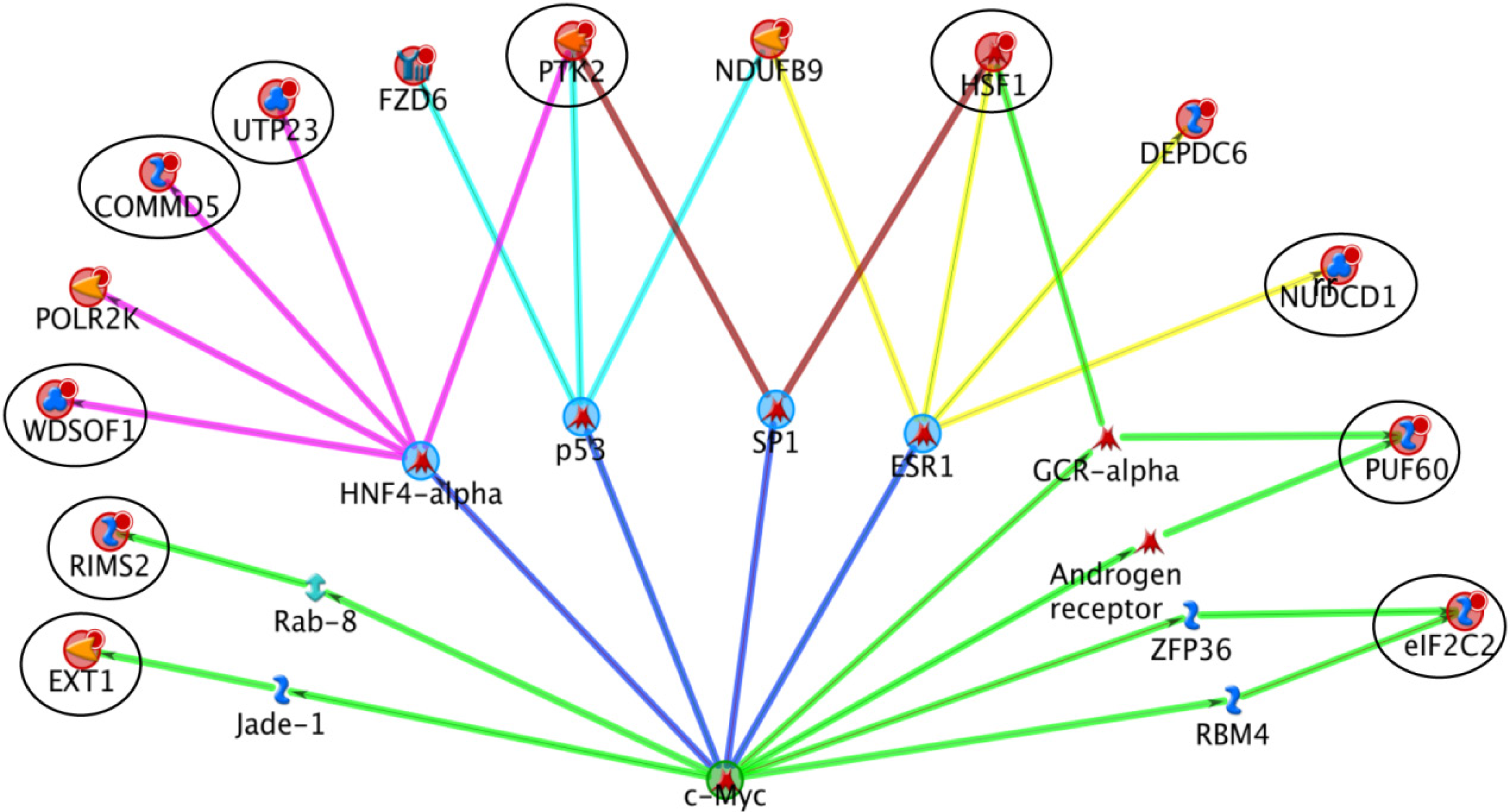

Supplement: Figure S3 — Nodes with red circles indicate the 14 genes influenced by cMyc through other transcription factors. Nodes with blue circles denote the top five master transcription factors other than cMyc. In our genetic component of gene expression analysis, the expression levels of 10 (black circles) of the 24 cis-control genes were predicted to be affected by the amplified cMyc locus. The results of genetic component of gene expression analysis were in agreement with enrichment analysis of transcriptional modules. (TIF) [file pone.0023452.s003.tif]
