# Supplementary material for: A Novel Molecular Signature Identified by Systems Genetics Approach Predicts Prognosis in Oral Squamous Cell Carcinoma
Source: PLoS One. 2011 Aug 11;6(8):e23452. doi: 10.1371/journal.pone.0023452 (PMC3154947; doi:10.1371/journal.pone.0023452)
Supplement: Table S1 — The 83 common CNVs. Each of these occurred in at least 40 OSCC patients. (DOC) [file pone.0023452.s004.doc]

**Table S1** The 83 common CNVs. Each of these occurred in at least 40 OSCC patients.

| **#chromosome** | **start** | **end** | **length (bps)** | **The number of  affected patients** | **Copy Number** |
| --- | --- | --- | --- | --- | --- |
| 6* | 103843095 | 103844700 | 1606 | 44 | Deletion |
| 6* | 103844700 | 103845062 | 363 | 45 | Deletion |
| 6* | 103845062 | 103854155 | 9094 | 46 | Deletion |
| 6* | 103854155 | 103854226 | 72 | 47 | Deletion |
| 6* | 103854226 | 103868733 | 14508 | 48 | Deletion |
| 6* | 103868733 | 103870458 | 1726 | 47 | Deletion |
| 7* | 142155261 | 142156316 | 1056 | 63 | Deletion |
| 7* | 142156316 | 142159174 | 2859 | 68 | Deletion |
| 7* | 142159174 | 142171879 | 12706 | 68 | Deletion |
| 8 | 101000476 | 101348744 | 348269 | 40 | Amplification |
| 8 | 101348744 | 102239988 | 891245 | 41 | Amplification |
| 8 | 102239988 | 102634362 | 394375 | 42 | Amplification |
| 8 | 102634362 | 104468714 | 1834353 | 43 | Amplification |
| 8 | 104468714 | 104473220 | 4507 | 42 | Amplification |
| 8 | 104473220 | 105045989 | 572770 | 43 | Amplification |
| 8 | 105045989 | 106820189 | 1774201 | 42 | Amplification |
| 8 | 106820189 | 106821898 | 1710 | 41 | Amplification |
| 8 | 106821898 | 109010618 | 2188721 | 42 | Amplification |
| 8 | 109010618 | 109574950 | 564333 | 43 | Amplification |
| 8 | 109574950 | 110357009 | 782060 | 44 | Amplification |
| 8 | 110357009 | 110915943 | 558935 | 45 | Amplification |
| 8 | 110915943 | 112268185 | 1352243 | 45 | Amplification |
| 8 | 112268185 | 112361991 | 93807 | 45 | Amplification |
| 8 | 112361991 | 112364235 | 2245 | 44 | Amplification |
| 8 | 112364235 | 112367432 | 3198 | 43 | Amplification |
| 8 | 112367432 | 112616078 | 248647 | 45 | Amplification |
| 8 | 112616078 | 115232457 | 2616380 | 46 | Amplification |
| 8 | 115232457 | 115594408 | 361952 | 45 | Amplification |
| 8 | 115594408 | 115698615 | 104208 | 43 | Amplification |
| 8* | 115698615 | 115702137 | 3523 | 42 | Amplification |
| 8 | 115715390 | 115766141 | 50752 | 41 | Amplification |
| 8 | 115766141 | 115889879 | 123739 | 42 | Amplification |
| 8 | 115889879 | 115932949 | 43071 | 43 | Amplification |
| 8 | 115932949 | 116984409 | 1051461 | 44 | Amplification |
| 8* | 116984409 | 117176089 | 191681 | 44 | Amplification |
| 8 | 117176089 | 117696416 | 520328 | 44 | Amplification |
| 8* | 117696416 | 117702319 | 5904 | 43 | Amplification |
| 8 | 117702319 | 119455900 | 1753582 | 44 | Amplification |
| 8 | 119455900 | 119717186 | 261287 | 43 | Amplification |
| 8 | 119717186 | 121345471 | 1628286 | 44 | Amplification |
| 8 | 121345471 | 122332581 | 987111 | 45 | Amplification |
| 8 | 122332581 | 123213482 | 880902 | 44 | Amplification |
| 8 | 123213482 | 123216039 | 2558 | 43 | Amplification |
| 8 | 123216039 | 123273590 | 57552 | 44 | Amplification |
| 8 | 123273590 | 126370411 | 3096822 | 45 | Amplification |
| 8 | 126370411 | 126702785 | 332375 | 44 | Amplification |
| 8 | 126702785 | 130680363 | 3977579 | 44 | Amplification |
| 8 | 130680363 | 131780976 | 1100614 | 45 | Amplification |
| 8 | 131780976 | 131783048 | 2073 | 44 | Amplification |
| 8 | 131783048 | 131810315 | 27268 | 45 | Amplification |
| 8 | 131810315 | 131883289 | 72975 | 46 | Amplification |
| 8 | 131883289 | 132626212 | 742924 | 45 | Amplification |
| 8* | 132626212 | 132627667 | 1456 | 44 | Amplification |
| 8 | 132627667 | 132946194 | 318528 | 45 | Amplification |
| 8 | 132946194 | 133298287 | 352094 | 44 | Amplification |
| 8 | 133298287 | 133713090 | 414804 | 43 | Amplification |
| 8 | 133713090 | 133924958 | 211869 | 43 | Amplification |
| 8 | 133924958 | 134447839 | 522882 | 42 | Amplification |
| 8 | 134447839 | 135128969 | 681131 | 41 | Amplification |
| 8 | 135128969 | 135140140 | 11172 | 40 | Amplification |
| 8 | 135140140 | 135147789 | 7650 | 41 | Amplification |
| 8 | 135147789 | 136053414 | 905626 | 40 | Amplification |
| 8 | 136053414 | 137068332 | 1014919 | 41 | Amplification |
| 8 | 137068332 | 137071085 | 2754 | 40 | Amplification |
| 8 | 137071085 | 138468510 | 1397426 | 41 | Amplification |
| 8 | 138468510 | 139583045 | 1114536 | 40 | Amplification |
| 8 | 139583045 | 141890601 | 2307557 | 41 | Amplification |
| 8 | 141890601 | 142075474 | 184874 | 42 | Amplification |
| 8 | 142075474 | 142231340 | 155867 | 41 | Amplification |
| 8 | 142231340 | 142586680 | 355341 | 40 | Amplification |
| 8 | 143461547 | 144770188 | 1308642 | 40 | Amplification |
| 8* | 144770188 | 144773300 | 3113 | 41 | Amplification |
| 8 | 144784148 | 144995051 | 210904 | 41 | Amplification |
| 8 | 144995051 | 145163601 | 168551 | 40 | Amplification |
| 8 | 145986967 | 146268960 | 281994 | 40 | Amplification |
| 11* | 55132965 | 55133080 | 116 | 41 | Amplification |
| 11* | 55133080 | 55209331 | 76252 | 42 | Amplification |
| 11* | 55209331 | 55209868 | 538 | 41 | Amplification |
| 12* | 9527156 | 9622897 | 95742 | 40 | Amplification |
| 17* | 36675887 | 36683691 | 7805 | 41 | Deletion |
| 17* | 36683691 | 36684016 | 326 | 41 | Deletion |
| 20* | 1509289 | 1541871 | 32583 | 44 | Amplification |
| 20* | 1541871 | 1543919 | 2049 | 43 | Amplification |

* CNVs reported in the Database of Genetic Variation.
