# Supplementary material for: A Novel Molecular Signature Identified by Systems Genetics Approach Predicts Prognosis in Oral Squamous Cell Carcinoma
Source: PLoS One. 2011 Aug 11;6(8):e23452. doi: 10.1371/journal.pone.0023452 (PMC3154947; doi:10.1371/journal.pone.0023452)
Supplement: Table S2 — Associations of the 24 amplified genes with different cancers according to published literature. (DOC) [file pone.0023452.s005.doc]

**Table S2** Associations of the 24 amplified genes with different cancers according to the published literature

| **Gene** | **Associated Cancers** | **Reference^a^** |
| --- | --- | --- |
| ***DEPDC6*** | multiple myelomas | (1) |
| ***LY6K*** | oral and breast cancer | (2-3) |
| ***LRP12*** | oral cancer | (4) |
| ***EXT1*** | salivary gland, oral, and gastroesophageal junction cancer | (5-7) |
| ***FZD6*** | prostate cancer, cutaneous squamous cell carcinoma, and pituitary adenomas | (8-9) |
| ***NUDCD1*** | leukemia, lung cancer, melanoma, and prostate cancer | (10-13) |
| ***MRPL13*** | oral cancer | (14) |
| ***POLR2K*** | oral cancer | (15-16) |
| ***PUF60*** | colon cancer and ovarian cancer | (17-18) |
| ***EIF2C2*** | oral cancer | (19) |
| ***NDUFB9*** | esophageal squamous cell carcinoma | (20) |
| ***UTP23*** | unknown | N/A |
| ***TAF2*** | esophageal squamous cell carcinoma, colon cancer | (21) |
| ***PYCRL*** | unknown | N/A |
| ***PTK2*** | oral, thyroid, breast and colon cancer | (19, 22-24) |
| ***GRINA*** | oral cancer | (25) |
| ***C8orf33*** | breast cancer | (26) |
| ***RIMS2*** | oral cancer | (27) |
| ***HSF1*** | oral cancer | (28) |
| ***MED30*** | breast cancer and follicular lymphoma | (29-30) |
| ***COMMD5*** | gastric cancer | (31) |
| ***ZNF707*** | prostate cancer | (32) |
| ***CHRAC1*** | oral, breast, prostate, and uterine cervical cancer | (19, 33-35) |
| ***DCAF13*** | breast cancer | (29) |

a1 Peterson, T.R., Laplante, M., Thoreen, C.C., Sancak, Y., Kang, S.A., Kuehl, W.M., Gray, N.S. and Sabatini, D.M. (2009) DEPTOR is an mTOR inhibitor frequently overexpressed in multiple myeloma cells and required for their survival. *Cell*, **137**, 873-886.

2 de Nooij-van Dalen, A.G., van Dongen, G.A., Smeets, S.J., Nieuwenhuis, E.J., Stigter-van Walsum, M., Snow, G.B. and Brakenhoff, R.H. (2003) Characterization of the human Ly-6 antigens, the newly annotated member Ly-6K included, as molecular markers for head-and-neck squamous cell carcinoma. *Int J Cancer*, **103**, 768-774.

3 Lee, J.W., Lee, Y.S., Yoo, K.H., Lee, K.H., Park, K., Ahn, T., Ko, C. and Park, J.H. (2006) LY-6K gene: a novel molecular marker for human breast cancer. *Oncol Rep*, **16**, 1211-1214.

4 Garnis, C., Coe, B.P., Zhang, L., Rosin, M.P. and Lam, W.L. (2004) Overexpression of LRP12, a gene contained within an 8q22 amplicon identified by high-resolution array CGH analysis of oral squamous cell carcinomas. *Oncogene*, **23**, 2582-2586.

5 van Duin, M., van Marion, R., Vissers, K.J., Hop, W.C., Dinjens, W.N., Tilanus, H.W., Siersema, P.D. and van Dekken, H. (2007) High-resolution array comparative genomic hybridization of chromosome 8q: evaluation of putative progression markers for gastroesophageal junction adenocarcinomas. *Cytogenet Genome Res*, **118**, 130-137.

6 Cromer, A., Carles, A., Millon, R., Ganguli, G., Chalmel, F., Lemaire, F., Young, J., with tumorigenesis and metastatic potential of hypopharyngeal cancer by microarray analysis. *Oncogene*, **23**, 2484-2498.

7 Frierson, H.F., Jr., El-Naggar, A.K., Welsh, J.B., Sapinoso, L.M., Su, A.I., Cheng, J., Saku, T., Moskaluk, C.A. and Hampton, G.M. (2002) Large scale molecular analysis identifies genes with altered expression in salivary adenoid cystic carcinoma. *Am J Pathol*, **161**, 1315-1323.

8 Haider, A.S., Peters, S.B., Kaporis, H., Cardinale, I., Fei, J., Ott, J., Blumenberg, M., Bowcock, A.M., Krueger, J.G. and Carucci, J.A. (2006) Genomic analysis defines a cancer-specific gene expression signature for human squamous cell carcinoma and distinguishes malignant hyperproliferation from benign hyperplasia. *J Invest Dermatol*, **126**, 869-881.

9 Miyakoshi, T., Takei, M., Kajiya, H., Egashira, N., Takekoshi, S., Teramoto, A. and Osamura, R.Y. (2008) Expression of Wnt4 in human pituitary adenomas regulates activation of the beta-catenin-independent pathway. *Endocr Pathol*, **19**, 261-273.

10 Yang, X.F., Wu, C.J., McLaughlin, S., Chillemi, A., Wang, K.S., Canning, C., Alyea, E.P., Kantoff, P., Soiffer, R.J., Dranoff, G. *et al.* (2001) CML66, a broadly immunogenic tumor antigen, elicits a humoral immune response associated with remission of chronic myelogenous leukemia. *Proc Natl Acad Sci U S A*, **98**, 7492-7497.

11 Wang, Q., Li, M., Wang, Y., Zhang, Y., Jin, S., Xie, G., Liu, Z., Wang, S., Zhang, H., Shen, L. *et al.* (2008) RNA interference targeting CML66, a novel tumor antigen, inhibits proliferation, invasion and metastasis of HeLa cells. *Cancer Lett*, **269**, 127-138.

12 Suemori, K., Fujiwara, H., Ochi, T., Azuma, T., Yamanouchi, J., Narumi, H., Yakushijin, Y., Hato, T. and Yasukawa, M. (2009) Identification of a novel epitope derived from CML66 that is recognized by anti-leukaemia cytotoxic T lymphocytes. *Br J Haematol*, **146**, 115-118.

13 Bianchi, F., Nuciforo, P., Vecchi, M., Bernard, L., Tizzoni, L., Marchetti, A., Buttitta, F., Felicioni, L., Nicassio, F. and Di Fiore, P.P. (2007) Survival prediction of stage I lung adenocarcinomas by expression of 10 genes. *J Clin Invest*, **117**, 3436-3444.

14 Chen, J., He, Q.Y., Yuen, A.P. and Chiu, J.F. (2004) Proteomics of buccal squamous cell carcinoma: the involvement of multiple pathways in tumorigenesis. *Proteomics*, **4**, 2465-2475.

15 Li, X.G., Li, N.Y., Liu, T.S., Zhang, S.Y. and Wang, Y.X. (2003) [Significance of VEGF expression and microvessel count in oral carcinoma]. *Shanghai Kou Qiang Yi Xue*, **12**, 123-126.

16 Na, X., Duan, H.O., Messing, E.M., Schoen, S.R., Ryan, C.K., di Sant'Agnese, P.A., Golemis, E.A. and Wu, G. (2003) Identification of the RNA polymerase II subunit hsRPB7 as a novel target of the von Hippel-Lindau protein. *EMBO J*, **22**, 4249-4259.

17 Tsuda, H., Birrer, M.J., Ito, Y.M., Ohashi, Y., Lin, M., Lee, C., Wong, W.H., Rao, P.H., Lau, C.C., Berkowitz, R.S. *et al.* (2004) Identification of DNA copy number changes in microdissected serous ovarian cancer tissue using a cDNA microarray platform. *Cancer Genet Cytogenet*, **155**, 97-107.

18 Matsushita, K., Tomonaga, T., Shimada, H., Shioya, A., Higashi, M., Matsubara, H., Harigaya, K., Nomura, F., Libutti, D., Levens, D. *et al.* (2006) An essential role of alternative splicing of c-myc suppressor FUSE-binding protein-interacting repressor in carcinogenesis. *Cancer Res*, **66**, 1409-1417.

19 Sparano, A., Quesnelle, K.M., Kumar, M.S., Wang, Y., Sylvester, A.J., Feldman, M., Sewell, D.A., Weinstein, G.S. and Brose, M.S. (2006) Genome-wide profiling of oral squamous cell carcinoma by array-based comparative genomic hybridization. *Laryngoscope*, **116**, 735-741.

20 Uchikado, Y., Inoue, H., Haraguchi, N., Mimori, K., Natsugoe, S., Okumura, H., Aikou, T. and Mori, M. (2006) Gene expression profiling of lymph node metastasis by oligomicroarray analysis using laser microdissection in esophageal squamous cell carcinoma. *Int J Oncol*, **29**, 1337-1347.

21 Rubie, C., Kempf, K., Hans, J., Su, T., Tilton, B., Georg, T., Brittner, B., Ludwig, B. and Schilling, M. (2005) Housekeeping gene variability in normal and cancerous colorectal, pancreatic, esophageal, gastric and hepatic tissues. *Mol Cell Probes*, **19**, 101-109.

22 Schneider, G.B., Kurago, Z., Zaharias, R., Gruman, L.M., Schaller, M.D. and Hendrix, M.J. (2002) Elevated focal adhesion kinase expression facilitates oral tumor cell invasion. *Cancer*, **95**, 2508-2515.

23 Owens, L.V., Xu, L., Dent, G.A., Yang, X., Sturge, G.C., Craven, R.J. and Cance, W.G. (1996) Focal adhesion kinase as a marker of invasive potential in differentiated human thyroid cancer. *Ann Surg Oncol*, **3**, 100-105.

24 Cance, W.G., Harris, J.E., Iacocca, M.V., Roche, E., Yang, X., Chang, J., Simkins, S. in benign and malignant human breast and colon tissues: correlation with preinvasive and invasive phenotypes. *Clin Cancer Res*, **6**, 2417-2423.

25 Choi, S.W., Park, S.Y., Hong, S.P., Pai, H., Choi, J.Y. and Kim, S.G. (2004) The expression of NMDA receptor 1 is associated with clinicopathological parameters and prognosis in the oral squamous cell carcinoma. *J Oral Pathol Med*, **33**, 533-537.

26 Vincent-Salomon, A., Lucchesi, C., Gruel, N., Raynal, V., Pierron, G., Goudefroye, R., Reyal, F., Radvanyi, F., Salmon, R., Thiery, J.P. *et al.* (2008) Integrated genomic and transcriptomic analysis of ductal carcinoma in situ of the breast. *Clin Cancer Res*, **14**, 1956-1965.

27 Lin, M., Morrison, C.D., Jones, S., Mohamed, N., Bacher, J. and Plass, C. (2009) Copy number gain and oncogenic activity of YWHAZ/14-3-3zeta in head and neck squamous cell carcinoma. *Int J Cancer*, **125**, 603-611.

28 Yamano, Y., Uzawa, K., Shinozuka, K., Fushimi, K., Ishigami, T., Nomura, H., Ogawara, K., Shiiba, M., Yokoe, H. and Tanzawa, H. (2008) Hyaluronan-mediated motility: a target in oral squamous cell carcinoma. *Int J Oncol*, **32**, 1001-1009.

29 Chin, S.F., Teschendorff, A.E., Marioni, J.C., Wang, Y., Barbosa-Morais, N.L., Thorne, N.P., Costa, J.L., Pinder, S.E., van de Wiel, M.A., Green, A.R. *et al.* (2007) High-resolution aCGH and expression profiling identifies a novel genomic subtype of ER negative breast cancer. *Genome Biol*, **8**, R215.

30 Weinkauf, M., Christopeit, M., Hiddemann, W. and Dreyling, M. (2007) Proteome- and microarray-based expression analysis of lymphoma cell lines identifies a p53-centered cluster of differentially expressed proteins in mantle cell and follicular lymphoma. *Electrophoresis*, **28**, 4416-4426.

31 Chen, B.L., Yu, J., Zeng, Z.R., Chu, W.K., Wong, C.Y., Cheng, Y.Y., Sung, J.J., Hu, P.J. and Leung, W.K. (2008) Rosiglitazone suppresses gastric carcinogenesis by up-regulating HCaRG expression. *Oncol Rep*, **20**, 1093-1097.

32 Nesslinger, N.J., Sahota, R.A., Stone, B., Johnson, K., Chima, N., King, C., Rasmussen, D., Bishop, D., Rennie, P.S., Gleave, M. *et al.* (2007) Standard treatments induce antigen-specific immune responses in prostate cancer. *Clin Cancer Res*, **13**, 1493-1502.

33 Naylor, T.L., Greshock, J., Wang, Y., Colligon, T., Yu, Q.C., Clemmer, V., Zaks, T.Z. and Weber, B.L. (2005) High resolution genomic analysis of sporadic breast cancer using array-based comparative genomic hybridization. *Breast Cancer Res*, **7**, R1186-1198.

34 Porkka, K.P., Tammela, T.L., Vessella, R.L. and Visakorpi, T. (2004) RAD21 and KIAA0196 at 8q24 are amplified and overexpressed in prostate cancer. *Genes Chromosomes Cancer*, **39**, 1-10.

35 Choi, Y.P., Kang, S., Hong, S., Xie, X. and Cho, N.H. (2005) Proteomic analysis of progressive factors in uterine cervical cancer. *Proteomics*, **5**, 1481-1493.
