# Supplementary material for: A Novel Molecular Signature Identified by Systems Genetics Approach Predicts Prognosis in Oral Squamous Cell Carcinoma
Source: PLoS One. 2011 Aug 11;6(8):e23452. doi: 10.1371/journal.pone.0023452 (PMC3154947; doi:10.1371/journal.pone.0023452)
Supplement: Table S3 — Cox regression analysis of different time-to-event clinical traits and the 24 selected genes, as well as interactions between the 24 genes. (DOC) [file pone.0023452.s006.doc]

**Table S3** Cox regression analysis of different time-to-event clinical traits and the 24 selected genes, as well as interactions between the 24 genes.

| **Risk factor** | **Significant Predictor** | **Hazard Ratio** | **P-value** |
| --- | --- | --- | --- |
| **Overall survival** | GRINA*PTK2 | 3.066 (1.277,7.365) ^a^ | 0.01 |
| **Disease-specific**  **survival** | NUDCD1*ZNF707  LRP12*PTK2  TAF2*WDSOF1  EIF2C2*RIM32  COMMD5*PYCRL | 40.963 (5.857, 286.496)  5.243 (2.422, 11.349)  0.002 (0.000, 0.057)  0.237 (0.088, 0.639)  0.011 (0.000, 0.83) | 0.000  0.000  0.000  0.004  0.041 |
| **Second primary tumors** | EIF2C2  EXT1*UTP23  C8orf33*ZNF707 | 370.632 (5.542, 24787.701)  0.000 (0.000, 0.064 )  166.750 (4.468, 6223.767) | 0.006  0.003  0.006 |
| **Local relapse** | MED30*UTP23  DEPDC6*GRINA | 389.169 (18.859, 8030.645)  2.259 (1.315, 3.880) | 0.000  0.003 |
| **Neck relapse** | DEPDC6*UTP23  C8orf33*MED30  DEPDC6*GRINA  NDUFB9*WDSOF1  PTK2*ZNF707 | 6.326 (1.459, 27.428)  24572.789 (126.399, 4777100.234)  2.312 (1.227, 4.355)  0.026 (0.003, 0.240)  15.784 (1.182, 210.732) | 0.014  0.000  0.010  0.001  0.037 |
| **Local relapse + neck relapse** | WDSOF1  C8orf33*MED30  DEPDC6*GRINA  NDUFB9*ZNF707  EIF2C2*NDUFB9  GRINA*LY6K  POLR2K*LY6K  EXT1*PTK2 | 0.004 (0.000, 0.065)  46157.118 (503.908, 4227915.461)  22.604 (5.914, 86.402)  62.789 (5.935, 664.462)  0.007 (0.001, 0.074)  12.568 (2.665, 59.275)  0.12 (0.025, 0.574)  12.479 (3.597, 43.290) | 0.000  0.000  0.000  0.001  0.000  0.001  0.008  0.000 |
| **Distant metastasis** | COMMD5*UTP23  MED30*PUF60  LRP12*ZNF707 | 0.001 (0.000, 0.256)  3497.986 (29.142, 419867.805)  5.479 (1.697, 17.692) | 0.014  0.001  0.004 |
| **Relapse (local + neck + distant metastases)** | DEPDC6  LRP12*MED30 | 1.418 (1.054, 1.909)  3.438 (1.773, 6.668) | 0.021  0.000 |

^a^ indicates the lower and upper bounds of the 95% confidence interval.
