# Supplementary material for: A Novel Molecular Signature Identified by Systems Genetics Approach Predicts Prognosis in Oral Squamous Cell Carcinoma
Source: PLoS One. 2011 Aug 11;6(8):e23452. doi: 10.1371/journal.pone.0023452 (PMC3154947; doi:10.1371/journal.pone.0023452)
Supplement: Table S4 — 85 CNV-associated genes. (DOC) [file pone.0023452.s007.doc]

**Table S4** 85 CNV-associated genes

| **Gene Symbol** | **Entrez Gene ID** | **Gene Name** |
| --- | --- | --- |
| AADACL1 | 57552 | neutral cholesterol ester hydrolase 1 |
| AHCYL2 | 23382 | adenosylhomocysteinase-like 2 |
| ARSJ | 79642 | arylsulfatase family, member J |
| ATXN7 | 6314 | ataxin 7 |
| BID | 637 | BH3 interacting domain death agonist |
| C12orf47 | 51275 | chromosome 12 open reading frame 47 |
| C14orf179 | 112752 | chromosome 14 open reading frame 179 |
| C16orf57 | 79650 | chromosome 16 open reading frame 57 |
| C16orf68 | 79091 | chromosome 16 open reading frame 68 |
| C3orf23 | 285343 | chromosome 3 open reading frame 23 |
| C5orf4 | 10826 | chromosome 5 open reading frame 4 |
| C8orf33 | 65265 | chromosome 8 open reading frame 33 |
| C8orf5 | 286046 | Kell blood group complex subunit-related family, member 6 |
| CDH8 | 1006 | cadherin 8, type 2 |
| CHCHD7 | 79145 | coiled-coil-helix-coiled-coil-helix domain containing 7 |
| CHRAC1 | 54108 | chromatin accessibility complex 1 |
| COMMD5 | 28991 | COMM domain containing 5 |
| CXCL5 | 6374 | chemokine (C-X-C motif) ligand 5 |
| CYC1 | 1537 | cytochrome c-1 |
| DAPK2 | 23604 | death-associated protein kinase 2 |
| DCAF13 | 25879 | WD repeats and SOF1 domain containing |
| DDIT3 | 1649 | DNA-damage-inducible transcript 3 |
| DEPDC6 | 64798 | DEP domain containing 6 |
| EFNA1 | 1942 | ephrin-A1 |
| EIF2C2 | 27161 | eukaryotic translation initiation factor 2C, 2 |
| EIF5A2 | 56648 | eukaryotic translation initiation factor 5A2 |
| ESM1 | 11082 | endothelial cell-specific molecule 1 |
| EXT1 | 2131 | exostoses (multiple) 1 |
| FAT1 | 2195 | FAT tumor suppressor homolog 1 (Drosophila) |
| FBXW4 | 6468 | F-box and WD repeat domain containing 4 |
| FYCO1 | 79443 | FYVE and coiled-coil domain containing 1 |
| FZD6 | 8323 | frizzled homolog 6 (Drosophila) |
| GGTA1 | 2681 | glycoprotein, alpha-galactosyltransferase 1 |
| GPD1L | 23171 | glycerol-3-phosphate dehydrogenase 1-like |
| GPR172A | 79581 | G protein-coupled receptor 172A |
| GRINA | 2907 | glutamate receptor, ionotropic, N-methyl D-aspartate-associated protein 1 (glutamate binding) |
| HSF1 | 3297 | heat shock transcription factor 1 |
| KAT2B | 8850 | K(lysine) acetyltransferase 2B |
| KIF13B | 23303 | kinesin family member 13B |
| LOC554223 | 554223 | hypothetical LOC554223 |
| LRP12 | 29967 | low density lipoprotein-related protein 12 |
| LY6K | 54742 | lymphocyte antigen 6 complex, locus K |
| MED27 | 9442 | similar to cofactor required for Sp1 transcriptional activation, subunit 8, 34kDa; mediator complex subunit 27; CRSP8 pseudogene |
| MED30 | 90390 | mediator complex subunit 30 |
| METTL7A | 25840 | methyltransferase like 7A |
| MRPL13 | 28998 | mitochondrial ribosomal protein L13 |
| MRPL15 | 29088 | mitochondrial ribosomal protein L15 |
| MTFR1 | 9650 | mitochondrial fission regulator 1 |
| MTP18 | 51537 | mitochondrial protein 18 kDa |
| NDUFB9 | 4715 | NADH dehydrogenase (ubiquinone) 1 beta subcomplex, 9, 22kDa |
| NDUFS8 | 4728 | NADH dehydrogenase (ubiquinone) Fe-S protein 8, 23kDa (NADH-coenzyme Q reductase) |
| NFIX | 4784 | nuclear factor I/X (CCAAT-binding transcription factor) |
| NRIP3 | 56675 | nuclear receptor interacting protein 3 |
| NUDCD1 | 84955 | NudC domain containing 1 |
| PBX1 | 5087 | pre-B-cell leukemia homeobox 1 |
| POLR2K | 5440 | polymerase (RNA) II (DNA directed) polypeptide K, 7.0kDa |
| PPM1L | 151742 | protein phosphatase 1 (formerly 2C)-like |
| PSMB5 | 5693 | proteasome (prosome, macropain) subunit, beta type, 5 |
| PTK2 | 5747 | PTK2 protein tyrosine kinase 2 |
| PUF60 | 22827 | poly-U binding splicing factor 60KDa |
| PYCRL | 65263 | pyrroline-5-carboxylate reductase-like |
| RBM15B | 29890 | RNA binding motif protein 15B |
| RBP1 | 5947 | retinol binding protein 1, cellular |
| REPIN1 | 29803 | replication initiator 1 |
| RIMS2 | 9699 | regulating synaptic membrane exocytosis 2 |
| RRAGD | 58528 | Ras-related GTP binding D |
| SDF2L1 | 23753 | stromal cell-derived factor 2-like 1 |
| SHARPIN | 81858 | SHANK-associated RH domain interactor |
| SLC16A3 | 9123 | solute carrier family 16, member 3 (monocarboxylic acid transporter 4) |
| SNRK | 54861 | SNF related kinase |
| SNRPB2 | 6629 | small nuclear ribonucleoprotein polypeptide B'' |
| SSSCA1 | 10534 | Sjogren syndrome/scleroderma autoantigen 1 |
| STC2 | 8614 | stanniocalcin 2 |
| TAF2 | 6873 | TAF2 RNA polymerase II, TATA box binding protein (TBP)-associated factor, 150kDa |
| TCEA3 | 6920 | transcription elongation factor A (SII), 3 |
| TMEM138 | 51524 | transmembrane protein 138 |
| TMEM206 | 55248 | transmembrane protein 206 |
| TMEM208 | 29100 | transmembrane protein 208 |
| TNFRSF12A | 51330 | tumor necrosis factor receptor superfamily, member 12A |
| TNFSF10 | 8743 | tumor necrosis factor (ligand) superfamily, member 10 |
| UTP23 | 84294 | UTP23, small subunit (SSU) processome component, homolog (yeast) |
| VPRBP | 9730 | Vpr (HIV-1) binding protein |
| VWA5A | 4013 | von Willebrand factor A domain containing 5A |
| WDYHV1 | 55093 | WDYHV motif containing 1 |
| ZNF707 | 286075 | zinc finger protein 707 |
