# Supplementary material for: A Novel Molecular Signature Identified by Systems Genetics Approach Predicts Prognosis in Oral Squamous Cell Carcinoma
Source: PLoS One. 2011 Aug 11;6(8):e23452. doi: 10.1371/journal.pone.0023452 (PMC3154947; doi:10.1371/journal.pone.0023452)
Supplement: Table S5 — Enrichment analyses of transcriptional modules and Gene Ontology processes for the 85 genes associated with the 16 high-confidence CNV regions. (DOC) [file pone.0023452.s008.doc]

**Table S5** Enrichment analyses of transcriptional modules and Gene Ontology processes for the 85 genes associated with the 16 high-confidence CNV regions.

| **Transcription Factor** | **Gene Ontology Processes** | **P^a^** | |
| --- | --- | --- | --- |
| ***HNF4-alpha*** | RNA metabolic process (2.708e04), RNA biosynthetic process (3.925e-04)^b^, transcription initiation from RNA polymerase II promoter (5.257e-04), establishment of tissue polarity (9.378e-04),cellular metabolic process (1.312e-03) | | 5.000E-37 |
| ***SP1*** | positive regulation of vasodilation (1.207e-04), positive regulation of cellular process (3.212e-04), regulation of vasodilation (3.272e-04), positive regulation of biological process (5.970e-04), negative regulation of nervous system development (7.581e-04) | 2.32E-31 | |
| ***MYC*** | peptidyl-lysine modification (3.977e-05), polyamine homeostasis (6.949e-04), B cell apoptosis (2.083e-03), hypusine metabolic process (2.777e-03), peptidyl-lysine modification to hypusine (2.777e-03) | 1.75E-28 | |
| ***p53*** | positive regulation of vasodilation (8.242e-05), apoptotic program (1.155e-04), release of cytochrome c from mitochondria (1.548e-04), activation of pro-apoptotic gene products (1.548e-04), regulation of vasodilation (2.237e-04) | 1.28E-25 | |
| ***ESR1* (nuclear)** | N-terminal peptidyl-lysine acetylation (2.273e-03), peptidyl-lysine acetylation (2.273e-03), positive regulation of retinoic acid receptor signaling pathway (2.273e-03), negative regulation of mitosis (3.407e-03), N-terminal protein amino acid acetylation (4.541e-03) | 9.21E-23 | |

^a,b^: The p-values in the third column are calculated for each transcription module, while the p-values in parentheses of column 2 are for each gene ontology term listed.
