# Supplementary material for: A Novel Molecular Signature Identified by Systems Genetics Approach Predicts Prognosis in Oral Squamous Cell Carcinoma
Source: PLoS One. 2011 Aug 11;6(8):e23452. doi: 10.1371/journal.pone.0023452 (PMC3154947; doi:10.1371/journal.pone.0023452)
Supplement: Table S6 — General characteristics of the study participants. (DOC) [file pone.0023452.s009.doc]

**Table S6** General characteristics of the study participants

| Characteristic | | Number of positives | Number of negatives |
| --- | --- | --- | --- |
| pN^a^ | ECS+^b^ | 42 | 40^c^ |
|  | ECS- | 30 |  |
| Second primary tumor | | 16 | 96 |
| Local relapse | | 29 | 83 |
| Neck lymph node relapse | | 28 | 84 |
| Distant metastasis | | 23 | 89 |

^a^ pN: lymph node metastasis

^b^ ECS: extracapsular spread

^c^ No information on ECS is available for patients without lymph node metastasis

Sex distribution: 102 males and 10 females.
